# Analysis of painful situations during unsedated esophagogastroduodenoscopy

**DOI:** 10.1055/a-2401-6804

**Published:** 2024-11-07

**Authors:** Hiromitsu Kanzaki, Sakiko Kuraoka, Takuya Satomi, Shotaro Okanoue, Kenta Hamada, Yoshiyasu Kono, Masaya Iwamuro, Seiji Kawano, Yoshiro Kawahara, Hiroyuki Okada, Motoyuki Otsuka

**Affiliations:** 1 92057 Gastroenterology and Hepatology, Okayama University Hospital, Okayama, Japan; 2 Internal medicine, Tsuyama Chuo hospital, Tsuyama, Japan; 3 12997 Practical Gastrointestinal Endoscopy, Okayama University, Okayama, Japan; 4 199491 Gastroenterology and Hepatology, Okayama University Graduate School of Medicine Dentistry and Pharmaceutical Sciences, Okayama, Japan; 5 36996 Internal medicine, Japanese Red Cross Society Himeji Hospital, Himeji, Japan

**Keywords:** Endoscopy Upper GI Tract, Quality and logistical aspects, Training, Quality management, Performance and complications

## Abstract

**Background and study aims** Although esophagogastroduodenoscopy (EGD) is a widely used technique, the procedure is often associated with discomfort. This study aimed to analyze painful situations, their frequency, and factors associated with patient discomfort during EGD.

**Patients and methods** This prospective observational study included patients scheduled to undergo EGD. Seven endoscopists recruited patients scheduled for EGD screening or surveillance. Each endoscopist enrolled 20 patients, performing 10 EGD procedures using ultraslim endoscopes and 10 with standard-sized endoscopes. Data regarding painful situations and frequency were collected using specialized buttons pressed by the patients during EGD. A survey about overall discomfort was conducted after the procedure.

**Results** We analyzed data from 140 patients. Esophageal insertion and duodenal observation were associated with the highest incidence of pressing the pain button, accounting for 59.3% and 40.7% of the cases, respectively. The factor associated with pressing the pain button during esophageal insertion was endoscopist experience (< 10 years). In contrast, younger age and female sex were the factors associated with pressing the pain button during duodenal observation. In the post-procedure survey, 63.6% of patients reported discomfort. Factors associated with patient discomfort included pressing the pain button during esophageal insertion (odds ratio [OR]: 2.84, *P* = 0.01) and previous painful EGD experience (OR: 2.41, *P* = 0.03).

**Concusions** This study provides objective data on painful situations, their frequency, and related factors during EGD. Further research and interventions focusing on pain reduction during endoscopic procedures are warranted. The results of this study will help endoscopists manage painful situations and potentially improve skills.

## Introduction

Esophagogastroduodenoscopy (EGD) is performed globally to diagnose patients with symptoms and screen or monitor upper gastrointestinal diseases. Gastric and esophageal cancers are the third and sixth leading causes of cancer-related deaths, respectively 1 . Early detection and treatment are crucial for preventing deaths associated with these cancers. According to several guidelines, surveillance EGD is recommended for high-risk patients with esophageal or gastric cancer 2 3 4 . Patients are recommended to undergo EGD every 6 months to yearly. Gastric cancer ranks second among all cancer incidences in Japan 5 . Therefore, EGD screening is recommended every 2 years as a health checkup for adults aged > 50 years 6 .

The two types of EGD are based on the insertion route. Transnasal endoscopy involves insertion of the endoscope through the nasal passage, resulting in less nausea and discomfort during the procedure than peroral endoscopy 7 . However, transnasal endoscopy requires use of an ultraslim endoscope. Moreover, it can sometimes be difficult to perform because of variations in the width of the nasal cavity caused by individual characteristics. Peroral endoscopy is the traditional method of performing EGD; however, it can cause various types of discomfort. Consequently, some patients require sedation during the procedures. Although sedation may contribute to patient satisfaction 8 9 10 11 , it is associated with a risk of adverse events, and patients and medical staff require time and effort for preparation and recovery. Moreover, sedation increases procedure costs 11 . Consequently, some patients have to endure discomfort without sedation. In addition, endoscope diameter has been suggested to be associated with patient discomfort, and ultraslim endoscopes may potentially reduce patient discomfort compared with standard-diameter endoscopes 12 13 14 15 . However, available data are insufficient to demonstrate their benefits.

To ensure patient satisfaction and prevent trauma during future examinations, EGD should be performed with minimal discomfort, even without sedation. Pain during EGD may be attributed to various factors, such as pharyngeal reflex and pressure build-up by sufflation of air in the digestive tract. However, no study has comprehensively addressed painful situations during EGD. Longer procedure times, fewer experienced endoscopists, and large endoscope diameters are thought to be associated with patient discomfort during EGD; however, only reports suggesting an association with endoscope diameters currently exist 12 13 14 15 . Such data are crucial for endoscopists to develop techniques that can effectively reduce patient discomfort. Furthermore, objective data are necessary to improve endoscopist training.

This prospective, exploratory, observational trial aimed to clarify the specific situations and frequency of pain experienced during EGD. We investigated patient subjective discomfort during EGD using a questionnaire. Furthermore, we performed an analysis to identify the associated factors.

## Patients and methods

### Study design

This prospective, exploratory, observational trial was conducted at Okayama University Hospital between September 2021 and March 2022. Asymptomatic patients who visited our hospital for screening or surveillance using EGD were enrolled. Prior to EGD, each patient provided written informed consent to participate in the study. Patients who underwent the EGD protocol were given a specialized button integrated with measuring software (Takei Scientific Instruments, Co., Ltd., Niigata, Japan) to measure pain, along with an attached biological information monitor. A post-procedure survey, including the patients and endoscopists, was conducted soon after the procedure. Specific situations, pain frequency during EGD, and associated factors were analyzed.

### Endoscopists

Seven endoscopists participated in this study, including four with more than 10 years of experience in EGD and three with 6 to 9 years of experience. Except for one, all endoscopists were certified by the Japan Gastroenterological Endoscopy Society.

### Participants

We enrolled patients who visited the Okayama University Hospital and underwent EGD. Inclusion criteria were as follows: the purpose of EGD was for screening or surveillance after endoscopic or radiational treatment of esophageal or gastric lesions; patients aged 20 to 80 years; and those who could voluntarily provide written informed consent. Exclusion criteria were as follows: patients with abdominal symptoms; patients who desired sedation during EGD; patients who had previously undergone surgical resection of the upper gastrointestinal tract; estimated EGD duration exceeding 10 minutes; patients with known lesions requiring treatment; patients who were pregnant or possibly pregnant; patients with Eastern Cooperative Oncology Group performance status > 3; and any other factors deemed inappropriate by the investigators. The endoscopists provided explanations to patients who met the eligibility criteria and registered them prospectively. Because previous studies indicated a correlation between endoscope diameter and pain 12 13 14 15 , each endoscopist examined 20 patients, with 10 patients each undergoing EGD with an ultraslim or standard-size endoscope. Endoscope type selection varied according to physician or patient preference. A total of 140 participants were included in this study.

### EGD protocol

**Fig. 1:**
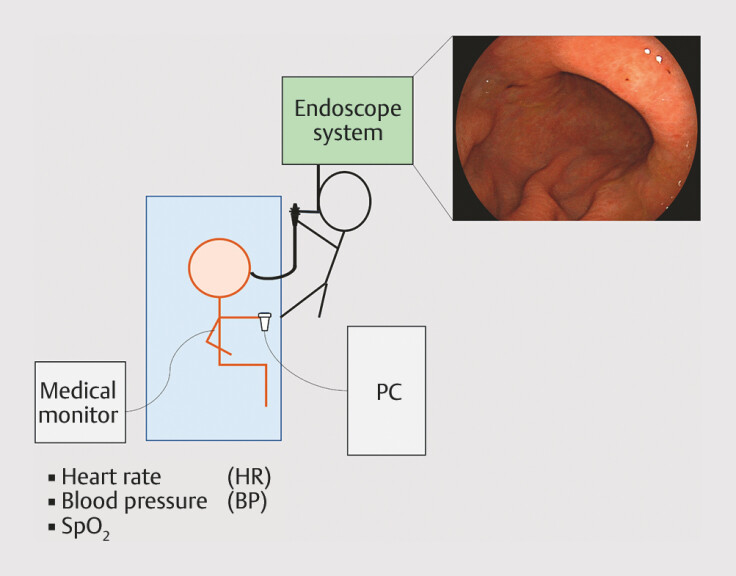
Esophagogastroduodenoscopy sites. A patient biological monitoring system, including percutaneous oxygen saturation, heart rate, and blood pressure monitors, was attached to patients before the procedure. Data were recorded before and during the procedure. The patients were instructed to press a button when they experienced pain. The button-press data were automatically recorded on a bedside personal computer using measurement software. The entire esophagogastroduodenoscopy procedure was recorded as video data in an institutional database. SpO _2_ ; percutaneous oxygen saturation, PC; personal computer.

The endoscopy system consisted of a light source (LL-7700; Fujifilm, Tokyo, Japan), a processor (VP-7000; Fujifilm), and a video monitor ( Fig. 1 ). The ultraslim endoscope was an EG-L580NW7 (Fujifilm, Tokyo, Japan), while the standard-size endoscope was an EG-L600ZW7 (Fujifilm, Tokyo, Japan). The diameters of the ultraslim and standard-size endoscopes were 5.8 mm and 9.9 mm, respectively. Endoscope type selection varied according to physician or patient preference. Approximately 5 minutes before the procedure, patients consumed a mixture of a mucolytic agent (20,000 U pronase, Pronase MS; Kaken Pharmaceutical Co., Ltd., Tokyo, Japan), 1 g sodium bicarbonate, and 3 mL dimethicone 2% internal solution (Fushimi Pharmaceutical Co. Ltd., Kagawa, Japan) diluted in 100 mL of tap water. Topical anesthesia was administered to the oropharynx using an 8% lidocaine spray (Xylocaine Pump Spray 8%; Sandoz Pharma K.K., Tokyo, Japan) immediately before EGD. A patient biological monitoring system, including percutaneous oxygen saturation (SpO _2_ ), heart rate (HR), and blood pressure (BP) monitors, was attached to the patients before the procedure. Data were recorded before and during the procedure. Intervals for recording SpO _2_ , HR, and BP were 1 minute, 1 minute, and 5 minutes, respectively. One nurse assisted the endoscopist during the EGD procedure. The nurse primarily functioned to support the endoscopist during procedures, such as biopsies. When they did not need to support the endoscopist, they gently touched the patient’s back to alleviate discomfort. Patients were instructed to press a button if they experienced any pain, including physical pain, nausea, or discomfort. They were instructed to keep pressing the button continuously during the pain and release it when the pain subsided. In addition, they were informed that they could press the button as many times as they felt pain during EGD. The button-press data were automatically recorded on a personal computer using measuring software. The entire EGD procedure was recorded as video data in an institutional database. Because there was no specific protocol for the EGD procedure, the endoscopists performed the procedure in their usual manner. Except for one, six endoscopists observed the esophagus, stomach, duodenum, and esophagus sequentially, whereas one endoscopist observed the esophagus, duodenum, stomach, and esophagus sequentially. All patients were surveyed for discomfort during EGD immediately after the procedure.

### Analysis of painful situations using button-press data

The button-press data consisted of the start and end times of each button press as well as the duration of each button press. Button-pressing situations were identified by reviewing the recorded EGD videos.

**Fig. 2:**
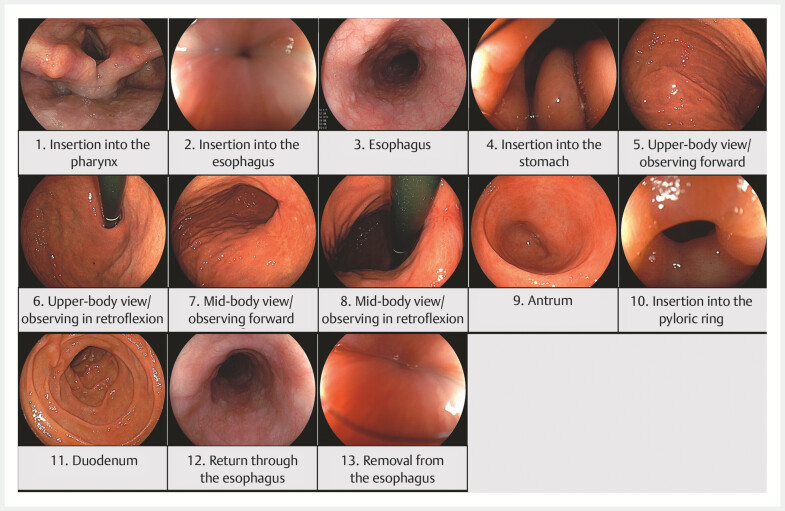
Painful situations. Button-pressing situations were divided into 13 categories for the analysis. These situations included insertion into the pharynx, insertion into the esophagus, observation within the esophagus, insertion into the stomach, upper-body view (observing forward and in retroflexion), mid-body view (observing forward and in retroflexion), antrum, insertion through the pyloric ring, observation of the duodenum, observation during return through the esophagus, and removal from the esophagus.

For the analysis, button-pressing situations were divided into 13 categories, including insertion into the pharynx, insertion into the esophagus, observation within the esophagus, insertion into the stomach, upper-body view, mid-body view (both observing forward and in retroflexion), antrum, insertion into the pyloric ring, observation of the duodenum, observation during return through the esophagus, and removal from the esophagus ( Fig. 2 ).

Even if the patient pressed the button for a short period, it was counted as the patient feeling discomfort. However, with such a definition, patients could continue to press the button during esophageal observation owing to discomfort from esophageal insertion, potentially leading to an overestimation of esophageal observations. Therefore, we decided to count esophageal insertion and observation only if the button was continuously pressed for more than 15 seconds from esophageal insertion to esophageal observation. However, if the button was released within 15 seconds, only the esophageal insertion would be counted.

### Background data from participants and procedure data from EGD

Characteristics of the study participants were collected prior to the procedure using a questionnaire. The registered data included sex, age, height, body weight, body mass index (BMI), ECOG-PS score, drinking and smoking habits, relationship with the assigned endoscopist, purpose of the EGD, previous EGD experience, previous painful EGD experience, time since last EGD, and discomfort level during last EGD.

EGD data included total procedure time, use of chromoendoscopy with iodine and indigo carmine dye, administration of antispasmodic drugs, whether a biopsy was performed, and histological assessment.

### Post-EGD survey

Patients underwent a survey immediately after EGD to assess whether they experienced discomfort during the examination. The Wong-Baker Faces Pain Rating Scale (0–5) was used to assess overall discomfort experienced, and patients with scores ≥ 2 were defined as experiencing discomfort.

### Outcomes

The primary objective of this study was to identify situations and frequency of patient pain, including physical pain, nausea, and any discomfort during EGD, by analyzing button-press data and differences in endoscope size. Other outcomes included factors associated with principal painful situations, factors associated with overall discomfort from the patient survey, and changes in biological data before and during EGD. These analyses were also conducted considering the endoscope size.

### Statistics

All statistical analyses were conducted using the JMP PRO software (ver. 15; SAS Institute Inc., Cary, North Carolina, United States). Continuous variables were expressed as medians with ranges. Univariate and multivariate logistic regression analyses were conducted to identify factors associated with pressing the pain button or experiencing discomfort. Age, BMI, BP, and HR were considered as continuous variables. Regarding duration of the EGD procedure, because a 7-minute threshold was considered 16 , the analysis was conducted by categorizing it as a nominal variable with a cutoff of 7 minutes. Statistical significance was set at *P* < 0.05.

## Results

To accumulate 140 cases, we surveyed a total of 150 patients; however, we excluded four patients who did not provide consent, and six were excluded during or after EGD, including four patients who had measurement failures, one aged > 80 years, and one who had previously undergone distal gastrectomy.

Table 1 shows characteristics of the 140 patients. Endoscope type selection varied based on physician or patient preference, resulting in differences in sex and age distributions. The ultraslim scope group comprised a higher proportion of young individuals and females. Two patients had tumorous lesions, one with a residual mucosa-associated lymphoid tissue lymphoma lesion and the other with a mucosal signet ring-cell carcinoma, first detected during this procedure.

**Table:** **Table 1** Participant background characteristics and procedure data.

	Total	Ultraslim	Standard size	*P* value
Sex (M/F)	71 (51)/69 (49)	29 (41)/41 (59)	42 (60)/28 (40)	0.042
Age (mean, range)	70 (21–79)	68 (21–79)	72 (33–79)	0.010
BMI kg/m ^2^ (mean, quadrant)	22.8 (20.5–25.1)	23.1 (20.6–25.2)	22.6 (20.5–25.0)	n.s.
ECOG PS (0/1/2)	130/9/1	67/3/0	63/6/1	n.s.
Alcohol consumption (none/past/few/much)	92/14/26/8	49/6/10/5	43/8/16/3	n.s.
Smoking (none/past/current)	76/46/18	40/22/8	36/24/10	n.s.
EGD experience (0/2–5 years/< 6 years)	3/47/90	1/27/42	2/20/48	n.s.
Interval since last EGD (first time/< 2 years/2–5 years/> 6 years)	3/110/24/3	1/51/15/3	2/59/9/0	n.s.
Relationship with the endoscopist (none/past EGD/periodic EGD)	119/15/6	61/6/3	58/9/3	n.s.
Sedation at last EGD (yes/no/first time)	17/120/3	8/61/1	9/59/2	n.s.
Previous painful EGD experience (yes/no/first time)	89/48/3	49/20/1	40/28/2	n.s.
Discomfort level of last EGD (none/mild to moderate/severe/first time)	63/56/18/3	31/26/12/1	32/30/6/2	n.s.
Purpose (surveillance/screening)	15/125	3/67	12/58	n.s.
Scope choice (physician/patient)	106 (76)/34 (24)	41 (59)/29 (41)	65 (93)/5 (7)	< 0.001
Stored images (mean, quadrant)				
Total	82 (71–95)	82 (72–93)	82 (68.8–98)	n.s.
Oropharynx	8 (6–12)	8 (6–11)	9 (7–12)	n.s.
Esophagus	15 (13–19)	15 (12–19)	15 (13–19)	n.s.
Stomach	53.5 (44–60)	54 (48–59)	53 (41–62)	n.s.
Duodenum	5 (4–6)	5 (4–6)	5 (3–6)	n.s.
Procedure time (seconds, mean, quadrant)	485.5 (410.8–594.5)	510.5 (425.5–600.8)	461 (384.3–547.5)	n.s.
Antispasmodic drugs	0	0	0	n.s.
Chromoendoscopy	62	30	32	n.s.
Biopsy	24	11	13	n.s.
Number of biopsies	1.5 (1–9)	1.5 (1–3)	1.5 (1–9)	n.s.
Tumorous lesion	2	2	0	n.s.
BMI, body mass index; ECOG PS, Eastern Cooperative Oncology Group performance status; EGD, esophagogastroduodenoscopy.				

### Situations and frequency of pressing the pain button during EGD

**Fig. 3:**
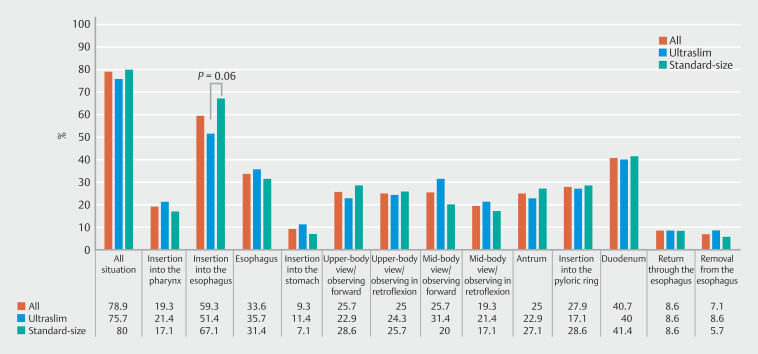
Situation and frequency of pain using button-pressing data. The overall button-pressing rate was 78.9%, indicating that 21.1% of patients did not press the button during esophagogastroduodenoscopy. The primary and secondary sites of pain were the esophagus (59.3%) and duodenum (40.7%), respectively. Although the ultraslim- and standard-size endoscopes showed no significant differences in any of the situations, there was a trend towards reduced pain during esophageal insertion with the ultraslim endoscope ( *P* = 0.06).

From the button-press data, 78.9% of patients (109/140) pressed the button at least once during the examination, whereas 21.1% (31/140) did not press the button. Among the 13 designated regions, 7.1% of patients (10/140) pressed the button in most regions (≥ 11). Among the designated 13 situations, the site with the highest pressing frequency was the esophagus (59.3%, 83/140), followed by the duodenum (40.7%, 57/140) ( Fig. 3 ). Although the difference was not significant, ultraslim endoscopy tended to be associated with a lower frequency of pressing the pain button during esophageal insertion ( *P* = 0.06).

Factors associated with pressing the pain button during esophageal insertion and duodenal observation differed ( Table 2 ). In the case of esophageal insertion, endoscopist experience played a role, whereas in duodenal observation, younger and female patients were more likely to press the pain button.

**Table:** **Table 2** Factors associated with pressing the pain button during esophageal insertion and duodenal observation.

		Odds ratio	95% CI	*P* value
**Esophageal insertion**				
Age	Per 1 year	0.98	0.95–1.01	0.22
EGD experience	> 6 times	1.40	0.70–2.83	0.34
Interval since last EGD	> 2 years	0.57	0.25–1.33	0.19
Sex	Male	1.11	0.57–2.19	0.76
Endoscope	Ultraslim	0.52	0.26–1.03	0.06
Endoscopist experience	> 10 years	0.4	0.19–0.81	0.01
Previous painful experience	+	1.78	0.87–3.63	0.11
Previous use of sedation	+	1.77	0.59–5.35	0.31
Smoking	+	1.13	0.58–2.23	0.72
Alcohol consumption	+	0.73	0.36–1.47	0.37
BMI (kg/m ^2)^	Per 1 up	1.00	0.92–1.08	0.98
**Duodenal observation**				
Age	Per 1 year	0.96	0.93–1.00	0.03
EGD experience	> 6 times	1.19	0.59–2.42	0.63
Interval since last EGD	> 2 years	0.55	0.22–1.36	0.20
Sex	Male	0.49	0.25–0.98	0.04
Endoscope	Ultraslim	0.94	0.48–1.85	0.86
Endoscopist experience	> 10 years	1.52	0.76–3.03	0.23
Previous painful experience	+	1.56	0.75–3.24	0.23
Previous use of sedation	+	1.05	0.37–2.95	0.93
Smoking habit	+	0.88	0.45–1.74	0.72
Alcohol consumption	+	0.71	0.35–1.47	0.36
BMI (kg/m ^2)^	Per 1 up	0.98	0.90–1.06	0.55
CI, confidence interval; EGD, esophagogastroduodenoscopy; BMI, body mass index.				

### Assessment of discomfort during EGD procedure using post-procedure survey

In post-EGD surveys, 63.6% of patients (89/140) reported discomfort, indicated by Wong-Baker Faces Pain Rating Scale scores ≥ 2. Patients who reported discomfort during EGD had painful experiences with previous EGD and pressed the button during esophageal insertion ( Table 3 ). However, longer procedure time and larger endoscope size, which were estimated to be correlated with discomfort, were not associated with patient discomfort.

**Table:** **Table 3** Factors associated with patient discomfort as reported on the survey.

		Univariate			Multivariate		
		Odds ratio	95% CI	*P* value	Odds ratio	95% CI	*P* value
Pre-procedure factor							
Age	per 1 year	0.98	0.95–1.01	0.23			
EGD experience	< 6 times	0.74	0.36–1.53	0.42			
Interval since last EGD	< 2 years	0.80	0.34–1.88	0.61			
Sex	Male	0.77	0.39–1.53	0.45			
Endoscope	Ultraslim	0.65	0.32–1.30	0.22			
Endoscopist	< 10 years	0.90	0.45–1.80	0.76			
Previous painful experience	+	2.63	1.27–5.45	0.01	2.41	1.12–5.22	0.03
Previous use of sedation	+	2.09	0.64–6.80	0.22			
Smoking	+	0.92	0.46–1.83	0.81			
Alcohol consumption	+	1.07	0.52–2.21	0.86			
BMI (kg/m ^2^ )	per 1 up	0.99	0.91–1.08	0.86			
Intra-procedure factor							
Procedure time	< 7 minutes	0.42	0.18–0.98	0.05	0.43	0.17–1.07	0.07
Biopsy	+	0.95	0.38–2.35	0.91			
Pressing the pain button							
Esophageal insertion	+	3.28	1.60–6.72	> 0.01	2.84	1.32–6.12	0.01
Duodenum	+	2.14	1.03–4.46	0.04	1.48	0.66–3.30	0.34
CI, confidence interval; EGD, esophagogastroduodenoscopy; BMI, body mass index.							

### Fluctuations in physiological data during EGD

Physiological data and changes before EGD, as well as maximum values during EGD, are presented in Table 4 . The differences in BP and HR before and at the maximum pain level were 5.5 mm Hg and 11.3 bpm, respectively. These changes in the discomfort group were significantly higher than those in the no-discomfort group (11.1 mm Hg vs. 1.1 mm Hg for BP and 14.7 bpm vs. 5.3 bpm for HR).

**Table:** **Table 4** Fluctuations in vital signs during EGD.

	**All** (n = 140)	**Discomfort** (n = 89)	**No discomfort** (n = 51)	***P* value **
**Blood pressure**				
Before EGD (Mean, quadrant)	142.6 (127 ~ 155)	140.1(124 ~154)	146.9(131 ~158)	0.09
Maximum during EGD (mean, quadrant)	150.1 (133 ~ 168.25)	151.3(134 ~169)	147.9(132.5 ~162.5)	0.49
Difference (mean, quadrant)	7.5 (-5 ~ 18)	11.1(0 ~20)	1.1(-8 ~6)	<0.01
**Heart rate**				
Before EGD (mean, quadrant)	72.5 (64.75 ~ 78)	72.8(64 ~79)	71.9(66 ~76)	0.64
Maximum during EGD (mean, quadrant)	83.7 (71 ~ 92.25)	87.5(74 ~100)	77.1(68.5 ~83)	< 0.01
Difference (mean, quadrant)	11.3 (2.75 ~ 16)	14.7(5 ~24)	5.3(0 ~8)	< 0.01
** SpO _2_**				
Before EGD (mean, quadrant)	97.5(96 ~99)	97.5(96 ~99)	97.3(96 ~98.5)	0.53
Maximum during EGD (mean, quadrant)	98.7(98 ~100)	98.8(98 ~100)	98.5(98 ~99)	0.17
Difference (mean, quadrant)	1.2(0 ~2)	1.3(0.5 ~2)	1.2(0 ~2)	0.67
EGD, esophagogastroduodenoscopy; SpO _2_ , percutaneous oxygen saturation.				

## Discussion

To the best of our knowledge, this study is the first to reveal painful situations during EGD, not through a questionnaire but by having patients use a self-push button during the procedure. In previous studies, investigations of discomfort during EGD relied on post-procedure questionnaires 12 13 14 15 , making it difficult to directly reflect the pain experienced by patients during the procedure. Using an objective method, we demonstrated the major painful situations during EGD, including esophageal insertion and duodenal observation. Although experienced endoscopists may already be aware of this through their practical experience, objective data were lacking prior to this study. In addition, we identified unique factors associated with each painful situation and patient objective discomfort, which would be of interest to clinical endoscopists.

Esophageal insertion is generally thought to be painful because the pharyngeal reflex is a biological reaction in humans. Hence, the result of its being the most painful situation was clear. However, the 59.3% button-pressing rate during esophageal insertion indicated that not all patients experienced pain in the area. Some patients were able to stand the esophageal insertion but experienced more pain after it. Duodenal observation was ranked second in the button-press data. Because the action of insertion into the pylorus is separate, this situation simply represents observation of the duodenum. Endoscopists should be aware that duodenal observation is a common cause of pain.

Intriguingly, discomfort-related factors during esophageal insertion and duodenal observation were distinct. The only significant factor associated with pressing the pain button during esophageal insertion was endoscopist experience. Esophageal insertion requires more technical expertise than other situations, and it is easy to understand that experience is the best teacher. However, factors influencing discomfort during duodenal observation included sex and age, which are issues that cannot be easily addressed through endoscopic techniques or scope modifications. Detection of duodenal neoplasia has increased in recent years, making duodenal observation increasingly important 17 18 . Although this study does not clarify which specific manipulation in the duodenum causes discomfort, it is crucial to recognize that female and younger patients are particularly susceptible to discomfort during duodenal observation, and this should be carefully considered during EGD.

The patient survey revealed an overall assessment of discomfort throughout the procedure. Patients who experienced discomfort during EGD reported significant previous experiences of discomfort during EGD and pain during esophageal insertion. The former may be due to personal characteristics or trauma and may be difficult to improve medically. However, pain during esophageal insertion was related to endoscopist experience and corresponded with endoscope diameter, which could be improved by the endoscopist gaining more experience or by feature development of an improved endoscope.

Factors associated with patient discomfort, such as scope size and longer procedure time, correlated significantly with patient discomfort. Regarding endoscope size, ultraslim endoscopes tended to demonstrate superiority during esophageal insertion ( *P* = 0.06), which is consistent with a previous report 12 . Similar trends in past reports have enhanced this fact. No noticeable differences were observed regarding longer procedure times. However, endoscopist characteristics could not be excluded, which could have led to bias ( Table 5 ). Therefore, we cannot conclude that reducing overall examination time alleviates patient discomfort.

**Table:** **Table 5** Endoscopist experience, procedure time, and patient-reported pain during esophageal insertion and discomfort.

Endoscopist	Endoscopy experience (years)	Procedure time (median, range)	Pain during esophageal insertion (%)	Discomfort with the questionnaire (%)
A	6	8.7 (5.8–14.3)	70 (14/20)	60 (12/20)
B	7	7.9 (4.7–12.0)	85 (17/20)	75 (15/20)
C	8	7.9 (5.6–15.1)	60 (12/20)	60 (12/20)
D	11	15.0 (10.4–27.7)	30 (6/ 20)	45 (9/20)
E	14	7.6 (5.6–9.7)	45 (9/20)	55 (11/20)
F	18	7.7 (5.7–13.2)	55 (11/20)	70 (14/20)
G	19	5.5 (3.8–10)	70 (14/20)	80 (16/20)

There were notable data regarding BP and HR during EGD. Changes in BP and HR before and during EGD were significant, and the differences were high among those who experienced discomfort during EGD. The physical burden of EGD is evident, and fluctuations in vital signs during the procedure are inevitable. However, significant fluctuations in vital signs during the examination can be considered risk factors for cardiovascular events. Therefore, endoscopists should be aware that vital signs fluctuate significantly, especially in patients experiencing discomfort.

This study has some limitations. First, it was an exploratory study primarily aimed at elucidating painful situations and their frequency during EGD. The sample size was not statistically significant, and there was no randomization concerning scope size and endoscopist. As a result, it was challenging to determine whether scope size affected patient discomfort. However, notably, patients with prior experience of painful EGD tended to opt for ultraslim endoscopy ( Table 1 ). Within this trend, the observed reduction in pain frequency with ultraslim endoscopes during esophageal insertion may have significant implications. Next, we defined 13 observation areas based on our experience. Nevertheless, we could not measure detailed conditions such as suction, air delivery, or scope compression. Finally, other factors may reduce patient pain, such as physician communication or the overall atmosphere, which may play a major role during EGD; however, we could not analyze these factors.

## Conclusions

This analysis provides valuable insights into specific pain situations during EGD. Understanding painful situations and contributing factors may help minimize discomfort. Further research and interventions aimed at reducing pain during endoscopic procedures are needed to improve patient satisfaction. Nonetheless, the results of this study can help endoscopists become aware of patient suffering as detailed in objective data.
